# Integrated bioinformatics analysis and experimental validation identified CDCA families as prognostic biomarkers and sensitive indicators for rapamycin treatment of glioma

**DOI:** 10.1371/journal.pone.0295346

**Published:** 2024-01-05

**Authors:** Ren Li, Yang Chen, Biao Yang, Ziao Li, Shule Wang, Jianhang He, Zihan Zhou, Xuepeng Li, Jiayu Li, Yanqi Sun, Xiaolong Guo, Xiaogang Wang, Yongqiang Wu, Wenju Zhang, Geng Guo

**Affiliations:** 1 Department of Neurosurgery, First Hospital of Shanxi Medical University, Taiyuan, Shanxi, China; 2 School of Public Health, Shanxi Medical University, Taiyuan, Shanxi, China; 3 Department of General and Vascular Surgery, Second Hospital of Shanxi Medical University, Taiyuan, Shanxi, China; 4 Department of Emergency, First Hospital of Shanxi Medical University, Taiyuan, Shanxi, China; Sapporo Ika Daigaku, JAPAN

## Abstract

The cell division cycle associated (CDCA) genes regulate the cell cycle; however, their relationship with prognosis in glioma has been poorly reported in the literature. The Cancer Genome Atlas (TCGA) was utilized to probe the CDCA family in relation to the adverse clinical features of glioma. Glioma single-cell atlas reveals specific expression of CDCA3, 4, 5, 8 in malignant cells and CDCA7 in neural progenitor cells (NPC)-like malignant cells. Glioma data from TCGA, the China Glioma Genome Atlas Project (CGGA) and the gene expression omnibus (GEO) database all demonstrated that CDCA2, 3, 4, 5, 7 and 8 are prognostic markers for glioma. Further analysis identified CDCA2, 5 and 8 as independent prognostic factors for glioma. Lasso regression-based risk models for CDCA families demonstrated that high-risk patients were characterized by high tumor mutational burden (TMB), low levels of microsatellite instability (MSI), and low tumor immune dysfunction and rejection (TIDE) scores. These pointed to immunotherapy for glioma as a potentially viable treatment option Further CDCA clustering suggested that the high CDCA subtype exhibited a high macrophage phenotype and was associated with a higher antigen presentation capacity and high levels of immune escape. In addition, hsa-mir-15b-5p was predicted to be common regulator of CDCA3 and CDCA4, which was validated in U87 and U251 cells. Importantly, we found that CDCAs may indicate response to drug treatment, especially rapamycin, in glioma. In summary, our results suggest that CDCAs have potential applications in clinical diagnosis and as drug sensitivity markers in glioma.

## Introduction

Glioma is the most prevalent cerebral malignancy among adults. It accounts for about 80% of entire malignancies. Glioma is characterized by an unfavorable prognosis and is prone to recurrence [1–3]. The median survival time in lower-grade glioma (LGG) is about 7 years; the 10-year survival rate is 47% [4, 5]. Glioblastoma multiforme is a type of glioma with invasiveness and MST of less than 2 years [6–8].

Currently, gliomas are conventionally treated with surgery, which can be combined with temozolomide or in some cases with low-intensity electric field adjuvant therapy [9, 10]. There are currently few drugs for the treatment of glioma, and progress in drug development in this area has been slow, as the physiological structure of the blood–brain barrier means that medicines are unable to effectively reach the tumor site [11, 12]. However, developments in nanotechnology offer opportunities to overcome this barrier, and improved progress is expected in the development of drug treatments for glioma [13]. Immunotherapy is different from conventional treatments and is currently the most promising new treatment for glioma [10, 14]. However, the immunosuppressive mechanism of glioma also presents a challenge to the progress of immunotherapy [15].

The CDCA family contains a total of eight genes. The current gene symbol for the gene encoding CDCA1 is NUF2 (NUF2 component of NDC80 kinetochore complex), which is participating in the composition of the NDC80 kinetochore complex [16, 17]. CDCA2 encodes a subunit of protein phosphatase 1, a cell-cycle associated protein, and targets it to chromatin during anaphase. Some studies have found that CDCA2 could stimulate colorectal cancer cell proliferation, as well as upregulate CCAD1 to facilitate the proliferation and migration of melanoma [18, 19]. CDCA3 is another protein-coding gene. KIF22 promotes bladder cancer further development by activating CDCA3 [20]. CDCA4 is a protein-coding gene. Studies have reported that CDCA4 is linked to progression of breast cancer, lung cancer and melanoma [21–23]. CDCA5 is a protein-coding gene. CDCA5 has been ascertained in multiple cancers and participates in the PI3K/AKT pathway to drive breast cancer development, as well as promote prostate cancer progression via effects on the ERK signaling pathway [24, 25]. Silencing CDCA5-induced apoptosis in prostate cancer through p53-p21 signaling [26]. CDCA6, also known as CBX2 (chromobox 2), promotes glioma cell proliferation and invasiveness by participating in Akt/PI3K pathway [27]. CBX2 affects the further development of gastric carcinoma through the YAP/beta-catenin pathway [28]. CDCA7 promotes lung cancer cell proliferation by modulating the cell cycle [29]. CDCA7 was reported to be connected to a mechanism related to inflammation of gastric cancer [30]. CDCA8 can suppress the proliferation and migration of glioblastoma [31]. CDCA8 is also important for estrogen-stimulated breast cancer cell proliferation [32]. However, there have been few studies focusing on CDCAs in glioma. Our study uses bioinformatics to analyze the important functions of CDCA2/3/4/5/7/8 in gliomas and provides insight that may help to guide clinical diagnosis and treatment.

## Materials and methods

### Data processing

To analyze dissimilarity in CDCA family level between normal and glioma samples, we extracted transcriptional profiles of 1137 GTEx normal samples and 689 TCGA glioma samples from UCSC (https://xenabrowser.net) [33]. All samples were processed using Toil [34]. Transcriptional profiles, clinical, somatic mutation, and microsatellite instability data of glioma samples for subsequent analyses were from TCGA (https://portal.gdc.cancer.gov/) [35]. Using the Perl programming language (https://www.perl.org), we extracted expression profiles in TPM (transcripts per million reads), clinical data and tumor mutational burden (TMB) of TCGA glioma samples and retained samples with complete survival information and clinical features. WHO grade, IDH status, 1p/19q co-deletion of TCGA cohort were from primary literature [36]. TCGA cohort served as the model training set. Transcript of 276 glioma samples for the model test set were from GEO (accession number: GSE16011; https://www.ncbi.nlm.nih.gov/geo/query/acc.cgi?acc=GSE16011). Clinical data of patients were derived from the original literature of GSE16011 [37]. RNA sequencing and clinical data for 693 and 325 glioma samples were from the CGGA database (dataset IDs: mRNAseq_693, mRNAseq_325; http://www.cgga.org.cn) as an additional test set [38–42]. We extracted consensus gene expression matrices for the four datasets from TCGA, GEO, and CGGA. To realize the clustering of glioma based on CDCA family, we combined the TCGA data set and two CGGA data sets. To model and remove data noise and obtain correct statistical inferences, we calibrated the four datasets via the R package “sva” [43].

### CDCA family expression and clinical traits correlation analysis

The data of CDCA family expression differential analysis were all log2 transformed. We performed the ROC analysis of the CDCA family in the above features via the R package “pROC”. All analysis results were visualized with the aid of the R package “ggplot2”.

### Single cell analysis

Glioma scRNA-seq, GSM3828673 of dataset GSE131928 were from GEO (https://www.ncbi.nlm.nih.gov/geo) [44]. We download single-cell expression matrix, UMAP dimensionality reduction and cell type metadata from Tumor Immunization Single Cell Center 2 (TISCH2) database (http://tisch.comp-genomics.org/) [45]. Dimensionality reduction and cell annotation of single-cell matrices were actualized via the R package “Seurat” [46]. UMAP visualization, violin plot, bubble plot, and differential gene analysis are all completed with the R package “Seurat”. Differential gene analysis was actualized in cell clusters with distinct expression patterns of the CDCA family. The threshold for differential genes were |LogFC| > 1 & adj.pvalue < 0.05. KEGG analysis was conducted via the R package “clusterProfiler”[47]. Histogram visualization was completed via R package “ggplot2”.

### Prognostic analysis of CDCA family

We divided the expression of CDCA family genes into two cohorts based on the median expression. Using the R package “survminer”, we performed Kaplan–Meier (KM) analyses of overall survival (OS), disease specific survival (DSS), progress free interval (PFI) between the high and low expression groups in TCGA cohort. And two CGGA cohorts and GEO cohort in OS were also been analyzed. COX regression analysis was actualized via R package “survival”.

### CDCA family prognostic model construction and validation

The optimal model was fitted via LASSO COX regression analysis by R package “glmnet” [48]. Using a penalty parameter evaluated through tenfold cross-validation, a criterion for the optimality and minimum penalty (λ) was chosen to avoid overfitting. Then, we calculated patients’ risk scores based on multivariate stepwise Cox regression analysis. The formula was as follows:

$$Risk\;score=\sum_{i}coeffient({CDCAs}_{i})*expression({CDCAs}_{i})$$

where *coefficient* and *expression* denote the regression coefficient and the normalized expression for each CDCA family gene, respectively. We divided training and validation samples into two cohorts according to the median risk score for subsequent analyses. R package “ggDCA” was used to actualize the decision curve analysis (DCA). Using the R package "pheatmap", we performed the risk profile analysis. R package "survminer" was used for KM survival analysis of the model on the training cohort and 3 exterior validation cohorts.

### Tumor mutational burden, microsatellite instability and tumor immune dysfunction and exclusion analyses

TMB data is divided into two CDCA cohorts using Perl. The R package “maftools” was employed to construct the waterfall charts. Tumor immune dysfunction and exclusion (TIDE) score was obtained from TIDE (http://tide.dfci.harvard.edu/) [49]. We accomplished the boxplot of TMB, MSI, and TIDE differences via the R package “ggplot2”. Survival analysis based on TMB, MSI, and TIDE was actualized via the R package “survival”. TMB, MSI, and TIDE were grouped using median scores.

### Clustering of gliomas samples based on CDCA family

With the R package “ConsensusClusterPlus”, we actualized consensus clustering on the dataset merged by TCGA and 2 CGGA cohorts [50]. We set the number of clusters from 2 to 9, then use the empirical cumulative distribution function to choose the best number of clusters. Principal component analysis was actualized via R package “stats”. R package “survminer” was used for KM survival analysis of clusters.

### Functional enrichment, gene set enrichment and variation analyses

Using the R package “limma” we actualized a differential expression analysis between the two clusters. Gene differential expression thresholds for functional enrichment analysis were set as false discovery rate (FDR)<0.001 and |log fold change (FC)|>2. Gene ontology (GO) analysis and gene set enrichment analysis (GSEA) were conducted using R packages “clusterProfiler,” “org.Hs.eg.db,” and “enrichplot”[47, 51, 52]. q-value and p-value thresholds of 0.05 were used for the GO enrichment analyses. All differentially expressed genes were used to perform GSEA to assess pathway differences in the MSigDB enrichment gene set (c2.cp.kegg.v7.4.symbols.gmt) between the two cluster. R package “GSVA” and “GSEABase” were used for gene set variation analysis (GSVA) [53, 54].

### Immune microenvironment analyses

Using R software and CIBERSORT (https://cibersortx.stanford.edu/), we assessed the immune landscapes and compared the proportion of 22 infiltrating immune cell types between the two clusters [55]. the process was reiterated 1000 times to ensure the stability of the findings. Discrepancies in the immune microenvironment discrepancy between two clusters were assessed via the R package “estimate” [56]. We contrasted gene expression levels of immune checkpoints, immunostimulator, major histocompatibility complex (MHC) molecules, chemokines, and chemokine receptors between two clusters and used R package “ggplot2” to construct boxplots to visualize the results. The immune checkpoint and MHC molecule genes were acquired from Charoentong’s research [57].

### Upstream regulation analysis of CDCAs

NetworkAnalyst database 3.0 (https://www.networkanalyst.ca/) was used to screen upstream microRNAs (miRNAs) that regulate CDCA expression [58]. LncBase 3.0 (https://diana.e-ce.uth.gr/lncbasev3) and miRNet 2.0 (https://www.mirnet.ca) were used to explore upstream associated LncRNAs that regulate the miRNAs [59, 60]. Relevant LncRNAs were selected using a Venn diagram.

### Culture of U87 and U251 cells

U87 and U251 cells were from Shanghai Institutes for Biological Sciences. All cells were maintained in Dulbecco’s modified Eagle medium (DMEM) (Hyclon) with 10% fetal bovine serum (FBS) (Gibco). Cells were cultured in a humidified incubator 37°C containing 5% CO_2_.

### miRNA overexpression

The mimics NC and hsa-miR-15b-5p were acquired from Genepharma (Shanghai, China). Mimics were transfected into glioma cells using RNAiMAX (Invitrogen). Forty-eight hours after transfection, cells were obtained, and total RNA was extracted by TRIzol® reagent (Invitrogen). Target gene expression after miRNA overexpression was detected by real-time quantitative PCR (RT-qPCR) analysis. The sequences of the mimics were as follows. NC sense: 5′-UUUGUACUACACAAAAGUACUG-3′; NC antisense: 5′-CAGUACUUUUGUGUAGUACAAA-3′. hsa-miR-15b-5p sense: 5′-UAGCAGCACAUCAUGGUUUACA-3′; hsa-miR-15b-5p antisense: 5′-UGUAAACCAUGAUGUGCUGCUA-3′.

### RT-qPCR

The total RNA extraction, reverse transcription, and RT-qPCR experiments were actualized in accordance with a previous protocol [61]. The following primers were used: CDCA2 forward, 5′-TGCCGAATTACCTCCTAATCCT-3′ and reverse, 5′-TGCTCTACGGTTACTGTGGAAA-3′; CDCA4 forward, 5′-GAGATGACGCAGGATGGGAC-3′ and reverse, 5′-TCCACGTCTGAGAACAGCTCT-3′; CDCA5 forward, 5′-GACGCCAGAGACTTGGAAATG-3’ and reverse, 5′-GGACCTCGGTGAGTTTGGAG-3′; CDCA7 forward, 5′-GGTCCCTTGACGCTCTACC-3′ and reverse, 5′-TGGGCGAATTATATGCGGAAG-3′; CDCA8 forward, 5′-GAAGGGCAGTAGTCGGGTG-3′ and reverse, 5′-TCACGGTCGAAGTCTTTCAGA-3′; β-actin forward, 5′-CATGTACGTTGCTATCCAGGC-3′ and reverse, 5′-CTCCTTAATGTCACGCACGAT-3′. The 2^−ΔΔ^CT method was utilized to calculate expression.

### Drug screening and experimental validation

GDSC (https://www.cancerrxgene.org/) is a drug information website and which contains drug reactions and sensitivities of cancer cells [62]. We used the website to obtain drug and cell line data, screened clinical drugs related to the CDCA family using R software, and evaluated drug sensitivity. And we will screen the drugs for further experiments to explore the effectiveness of the drugs. IC50 assay was evaluated by the CCK-8 method (Beyotime, China). To assess cell sensitivity to the Rapamycin (HY-10219, MCE), U87 and U251 cells were cultured with or without Rapamycin for 48 hours, followed by the addition of 10 μl CCK-8 reagent and incubation for 2 h. The absorbance of each well at 450 nm was measured on a microplate reader (HBS-1096, DieTie, Nanjing, China).

### Statistical analysis

All statistical analyzes were performed using R software (V4.1.2). The normality of the glioma expression data was tested via the Shapiro-Wilk normality test. Since the expression data of glioma samples didn’t fit the normal distribution, we used Mann-Whitney U test to test the expression differences between the two cohorts of samples, and used the Dunn’s test that corrected the significance level by the Bonferroni method to perform intra-group comparison of 3 or more groups of samples. Statistical tests for survival analysis were conducted via proportional hazards model. The Spearman method was used for correlation analyses and to calculate correlation coefficients. pvalue<0.05 was considered to be statistically significant. Significance markers: ns, p≥0.05; *, p< 0.05; **, p<0.01; ***, p<0.001.

## Result

### CDCA family is interrelated with worse clinical traits of gliomas

**Fig 1:**
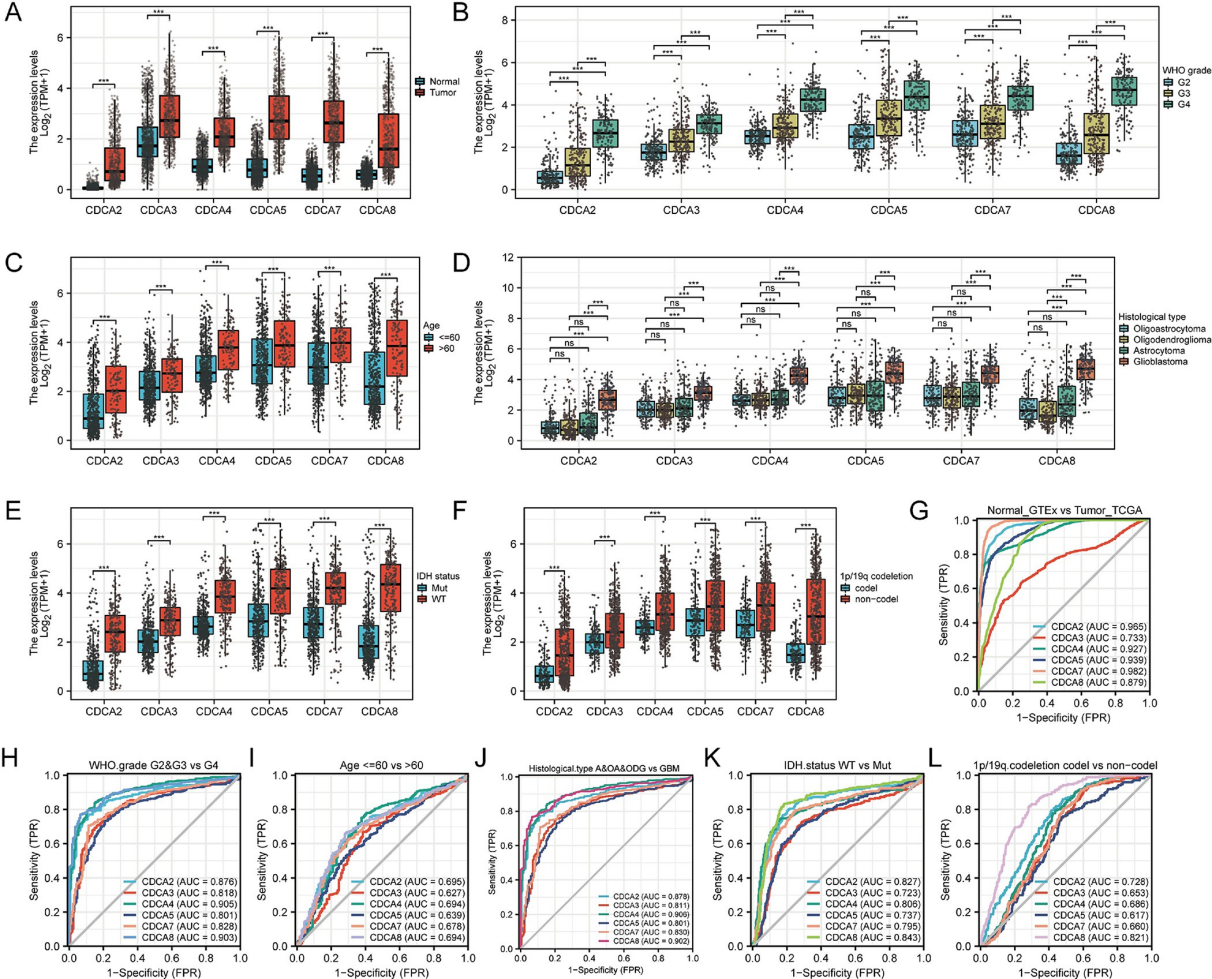
Cell division cycle associated family is associated with worse clinical characteristics of gliomas. **(A)** The mRNA levels of CDCAs in glioma tissues were significantly highly expressed. **(B)** In the WHO grade, the mRNA expression level of CDCAs was elevated as the WHO grade increased. **(C)** The mRNA expression levels of CDCAs were significantly higher in patients over 60 years of age. **(D)** In the histological type of glioma, CDCAs were significantly highly expressed in glioblastoma. **(E)** CDCAs were significantly highly expressed in IDH wild type. **(F)** CDCAs were highly expressed in the non-coding group of the 1p/19q codeletion state. Significance markers: ns, p≥0.05; *, p< 0.05; **, p<0.01; ***, p<0.001. **(G-L)** Predictive power of CDCA in diagnosing tumors and distinguishing outcomes with different clinical features. The horizontal coordinate represents 1-Specificity (FPR), which is the false positive rate, the larger the FPR, the higher the predicted false positive rate. The vertical coordinate represents Sensitivity (TRP), which represents the true positive rate.

We used glioma transcriptome data from the TCGA database and normal brain data from the GTEx database to explore the association between the CDCA family and clinical features of gliomas. The mRNA expression levels of CDCAs were further investigated in gliomas **(Fig 1A)**. CDCA2, CDCA3, CDCA4, CDCA5, CDCA7, and CDCA8 were significantly highly expressed in glioma samples. The mRNA level of CDCA2 was the lowest and CDCA3 and 5 were the highest. Then the interrelationship between clinical traits and the expression of CDCAs was analyzed. The expression of CDCAs increases with increasing World Health Organization (WHO) grades, they were the highest in G4 and the lowest in G2 **(Fig 1B)**. In addition, CDCAs were more highly expressed in the cohort of patients older than 60 years **(Fig 1C)**. CDCAs have higher expression in glioblastoma than other histological types **(Fig 1D)**. CDCAs expression was higher in wild-type glioma than in IDH-mutant type **(Fig 1E)**. CDCAs showed lower expression in the 1p/19q codeletion cohort compared with the non-codeletion cohort **(Fig 1F)**. Moreover, we analyzed the diagnostic ability of CDCAs based on clinical characteristics of patients with glioma. CDCA2, CDCA4, CDCA5, CDCA7, and CDCA8 showed high accuracy for diagnosing gliomas, with CDCA7 having the highest accuracy (AUC: 0.982) **(Fig 1G)**. CDCAs had favorable ability to predict WHO grades (all the AUC > 0.8) **(Fig 1H)**. CDCAs had some ability to predict patients’ ages **(Fig 1I)**. They had favorable ability in predicting histological types (all the AUC > 0.8) **(Fig 1J)**. CDCA2, CDCA4 and CDCA8 had favorable ability in predicting IDH status (all the AUC > 0.8) **(Fig 1K)**. Regarding 1p/19q codeletion, only CDCA8 showed high accuracy (AUC = 0.821) **(Fig 1L)**. The CDCA family has demonstrated high clinical value in the diagnosis of gliomas and in detecting different clinical traits.

### CDCA family Specifically expressed in malignant cells and neural progenitor cells (NPC)-like malignant cells

**Fig 2:**
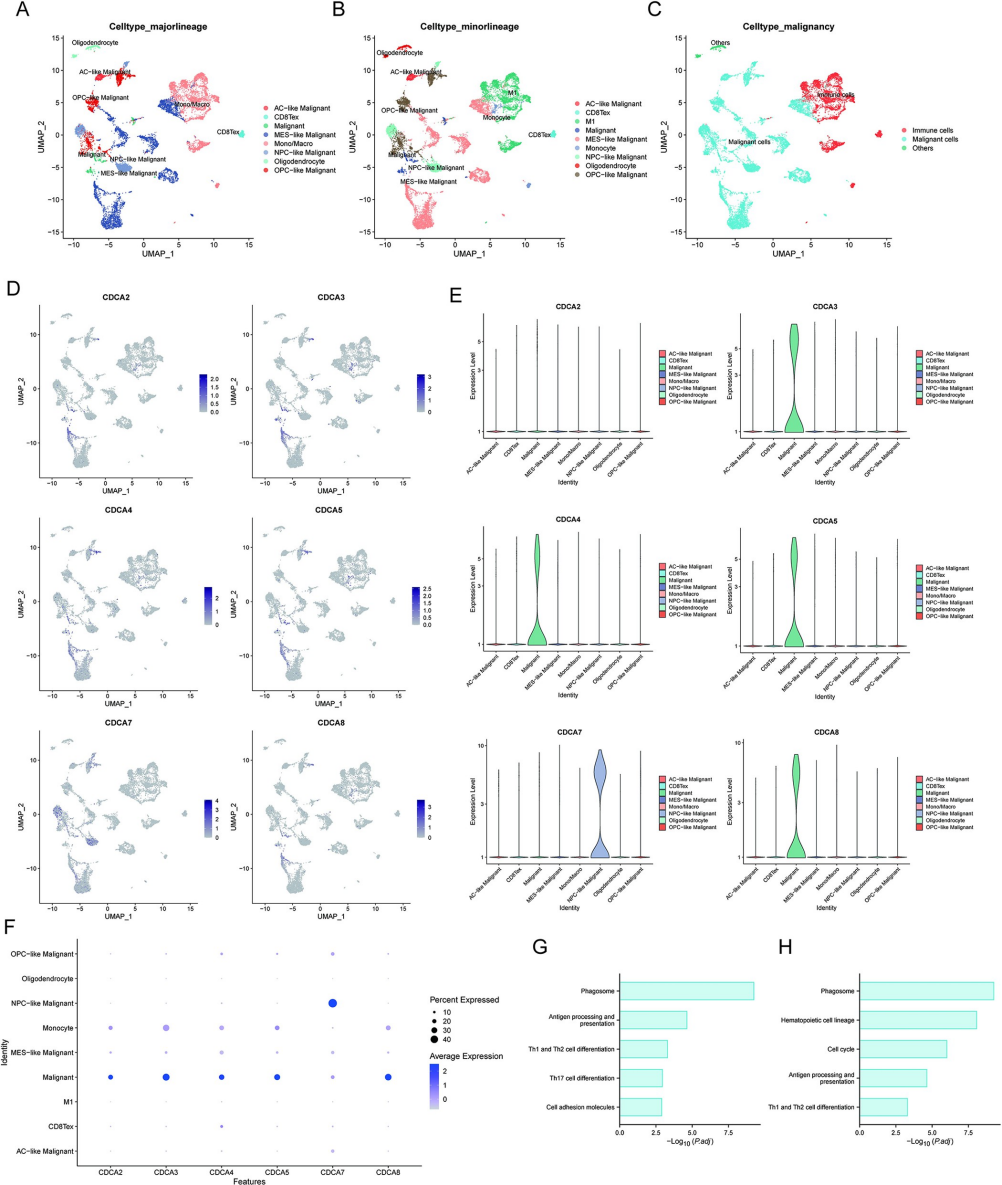
Single-cell transcriptional atlas of the CDCA family. **(A-C)** UMAP visualization of glioma. Color codes the cell types at the major-lineage **(A)**, minor-lineage **(B)** and malignancy **(C)** in turn. **(D)** UMAP visualization of CDCA family. **(E)** Violin plots of CDCA family at major-lineage cell type. And color codes the cell types at the major-lineage. **(F)** Bubble plot of CDCA family at minor-lineage cell type. **(G)** KEGG enrichment analysis of malignant cell clusters with CDCA3, 4, 5, 8 highly expressed. **(H)** KEGG enrichment analysis of NPC-like malignant cell clusters with CDCA7 intensively expressed.

We revealed CDCAs expression characteristics using the glioma single-cell atlas. UMAP visualization showing cell clusters in glioma tissue at major-lineage **(Fig 2A)**, minor-lineage **(Fig 2B)**, and malignancy level **(Fig 2C)**. The major-lineage included AC-like malignant, MES-like malignant, NPC-like malignant, OPC-like malignant, CD8Tex, malignant, CD8Tex, oligodendrocyte, and mono/macro. The minor-lineage refines Mono/Macro to M1 and Monocyte. CDCA2, 3, 4, 5, 8 had similar expression patterns, while the expression pattern of CDCA7 was specific **(Fig 2D)**. Further violin plots revealed that CDCA3, 4, 5, 8 are distinctively expressed in malignant cells, while CDCA7 was specifically expressed in NPC-like malignant cells **(Fig 2E)**. The bubble plot verified the expression pattern of the CDCA family at the minor-lineage **(Fig 2F)**. Malignant cells with CDCA3, 4, 5, 8 specifically expressed were enriched in phagosome, hematopoietic cell lineage and cell cycle **(Fig 2G)**. NPC-like malignant cells with CDCA7 specifically expressed were mainly enriched in phagosome, antigen processing, and presentation **(Fig 2H)**.

### The CDCA family showed significant prognostic value in all four datasets of TCGA, CGGA and GEO

**Fig 3:**
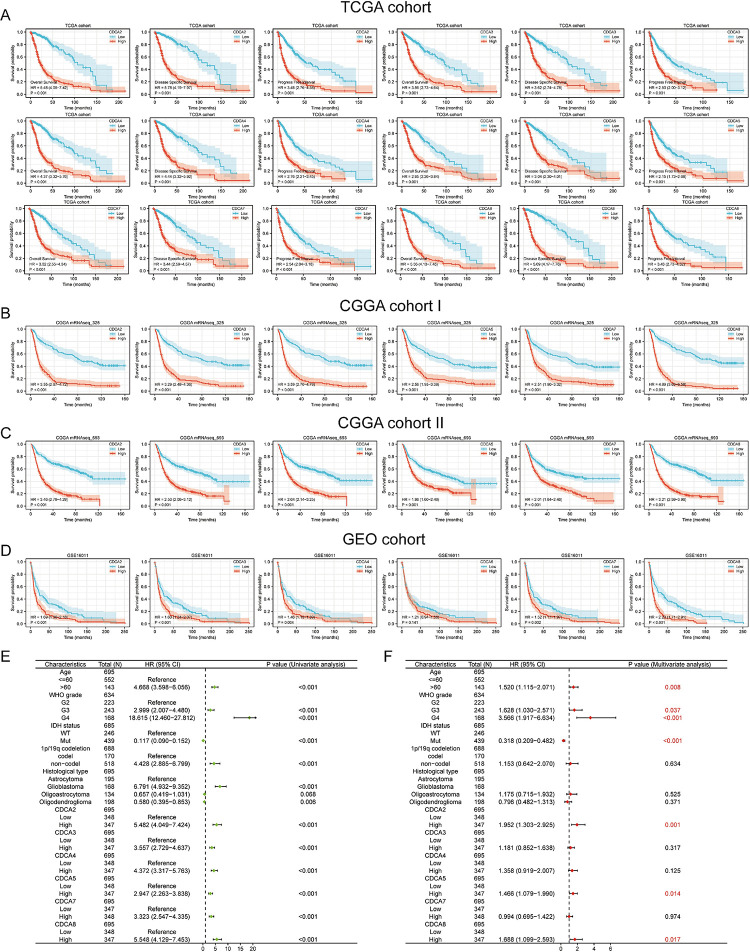
Cell division cycle associated family has an important prognostic value in glioma. **(A)** In the TCGA cohort, high CDCAs expression was associated with a lower survival rate. The TCGA prognostic evaluation includes three important parameters, which are overall survival, disease free survival and progression free interval. **(B)** Prognostic value of CDCAs in the mRNAseq_325 cohort of the Chinese glioma genome atlas with the parameter Overall Survival. **(C)** Prognostic value of CDCAs in the mRNAseq_693 cohort of the Chinese glioma genome atlas with the parameter Overall Survival. **(D)** Prognostic value of CDCAs in the GSE16011 cohort of the GEO public dataset with the parameter Overall Survival. **(E-F)** COX regression analysis of CDCAs in the TCGA cohort. Multi-factor COX regression analysis showed that CDCA2, CDCA5 and CDCA8 could be used as independent prognostic factors for glioma. P value<0.05 was considered statistically significant.

KM survival analysis was conducted on the TCGA, 2 CGGA, and GEO cohorts **(Fig 3A–3D)**. In TCGA cohort, patients with higher CDCAs level had worse OS, DSS, and PFI (p < 0.001) **(Fig 3A)**. Higher CDCAs heralded worse OS in CGGA _325 cohort **(Fig 3B)** and CGGA_693 cohort (p<0.001) **(Fig 3C)**. In GEO cohort, patients with higher level of CDCA2, CDCA3, CDCA4, CDCA7, and CDCA8 had worse OS **(Fig 3D)**. CDCA5 has no prognostic value in the GEO cohort (p = 0.141). We actualized COX regression analyses to examine the predictive power of CDCA family in glioma **(Fig 3E and 3F)**. Univariate COX regression analysis presented that all the CDCAs were the risk factors for glioma patients **(Fig 3E)**. And subsequent multivariate COX regression analyses suggested that CDCA2, CDCA5, and CDCA8 were independent prognostic factors for glioma **(Fig 3F)**.

### Prognostic effect of CDCA family combinations was found to be better than that of individual gene

**Fig 4:**
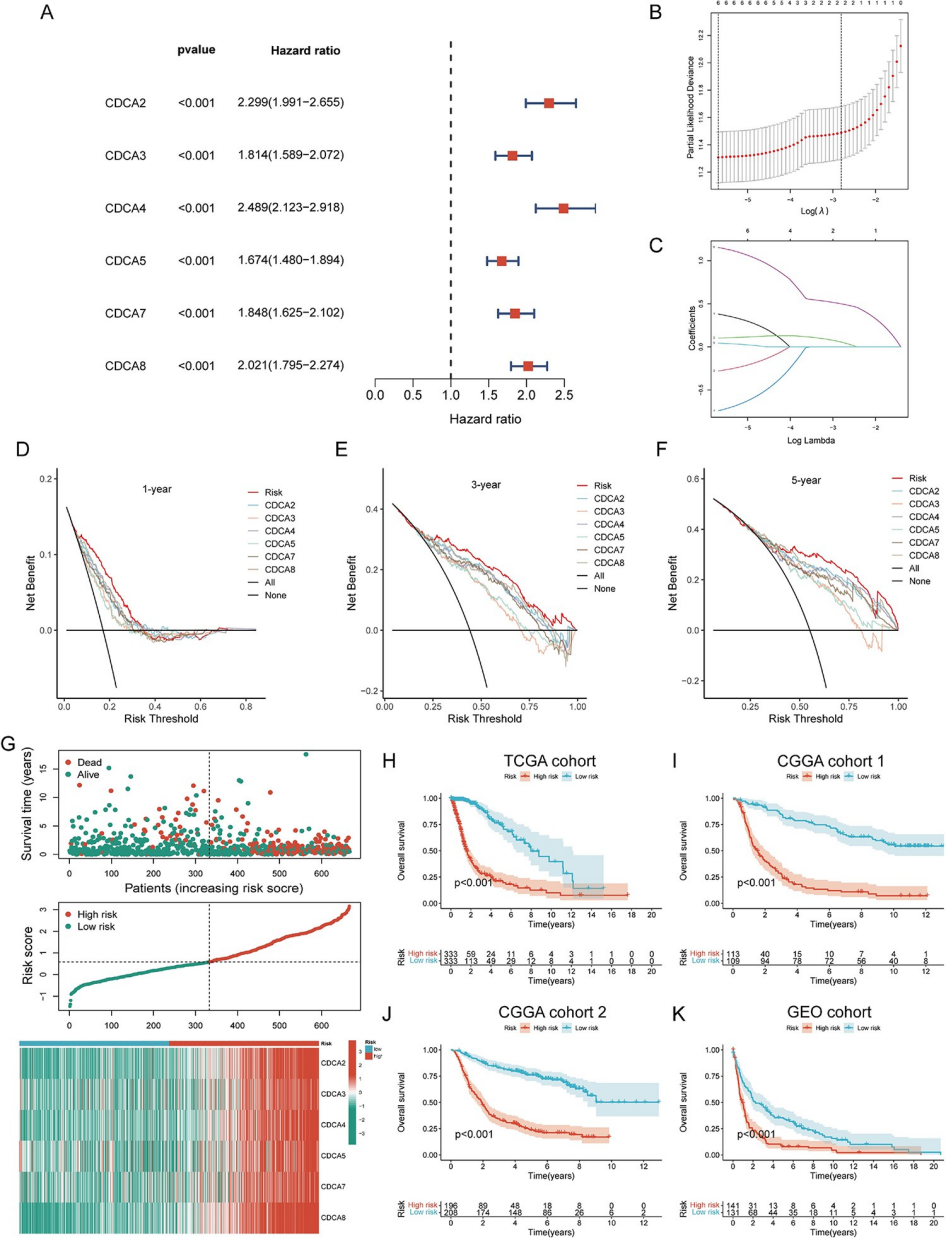
The prognostic efficacy of the combination of cell division cycle associated family is better than the prognostic efficacy of individual genes. **(A)** A univariate COX regression analysis of risk evaluates the riskiness of CDCAs. **(B-C)** Lasso regression analysis was performed to screen risk model variables, and the data were cross-validated using ten-fold. **(D-F)** The decision curve analysis found that the clinical utility of the risk model was better than that of a single CDCA at 1, 3 and 5 years. The x-axis represents the probability threshold and the y-axis represents the net revenue. **(G)** Risk factor plots demonstrated that high-risk patients were associated with worse clinical survival. **(H)** Kaplan-Meier curves showed that high-risk patients had a lower probability of survival. **(I-K)** CGGA’s datasets mRNAseq_325, mRNAseq_693 and GEO’s dataset GSE16011 were used as external data validation sets to verify the prognostic value of the risk model in glioma patients.

By univariate COX regression analysis, we confirmed that all CDCAs are prognostic risk factors for glioma **(Fig 4A)**. We then fitted a risk prognostic model of CDCAs by LASSO algorithm using TCGA training set to further analyze the prognostic role of CDCAs with glioma patients **(Fig 4B and 4C)**. And the formula was: risk score = (0.3810) * CDCA2 + (-0.2793) * CDCA3 + (0.1024) * CDCA4 + (-0.7410) * CDCA5 + (0.0437) * CDCA7 + (1.1541) * CDCA8. To compare the predictive accuracy of the CDCAs model and individual CDCAs genes in glioma prognosis, we performed DCA analysis **(Fig 4D–4F)**. The predictive gains of CDCAs model in 1 **(Fig 4D)**, 3 **(Fig 4E)**, and 5 years **(Fig 4F)** prognosis of glioma are higher than that of individual CDCAs genes. We performed prognostic analyses of the CDCA model in TCGA training cohort and CGGA and GEO validation cohorts **(Fig 4G–4K)**. The risk curve further indicated that the CDCA model predicted worse outcome in the high-risk cohort, and the risk heatmap indicated that all CDCAs genes were up-regulated in the high-risk cohort **(Fig 4G)**. High-risk patient cohorts had worse prognosis in TCGA **(Fig 4H)**, CGGA_325 **(Fig 4I)**, CGGA_693 **(Fig 4J)**, GEO datasets (p<0.001) **(Fig 4K)**. The combined predictive ability of the CDCA family is superior to that of individual CDCA genes and will have a stronger clinical application.

### The high CDCAs cohort was characterized by high TMB, low MSI, and low TIDE scores

**Fig 5:**
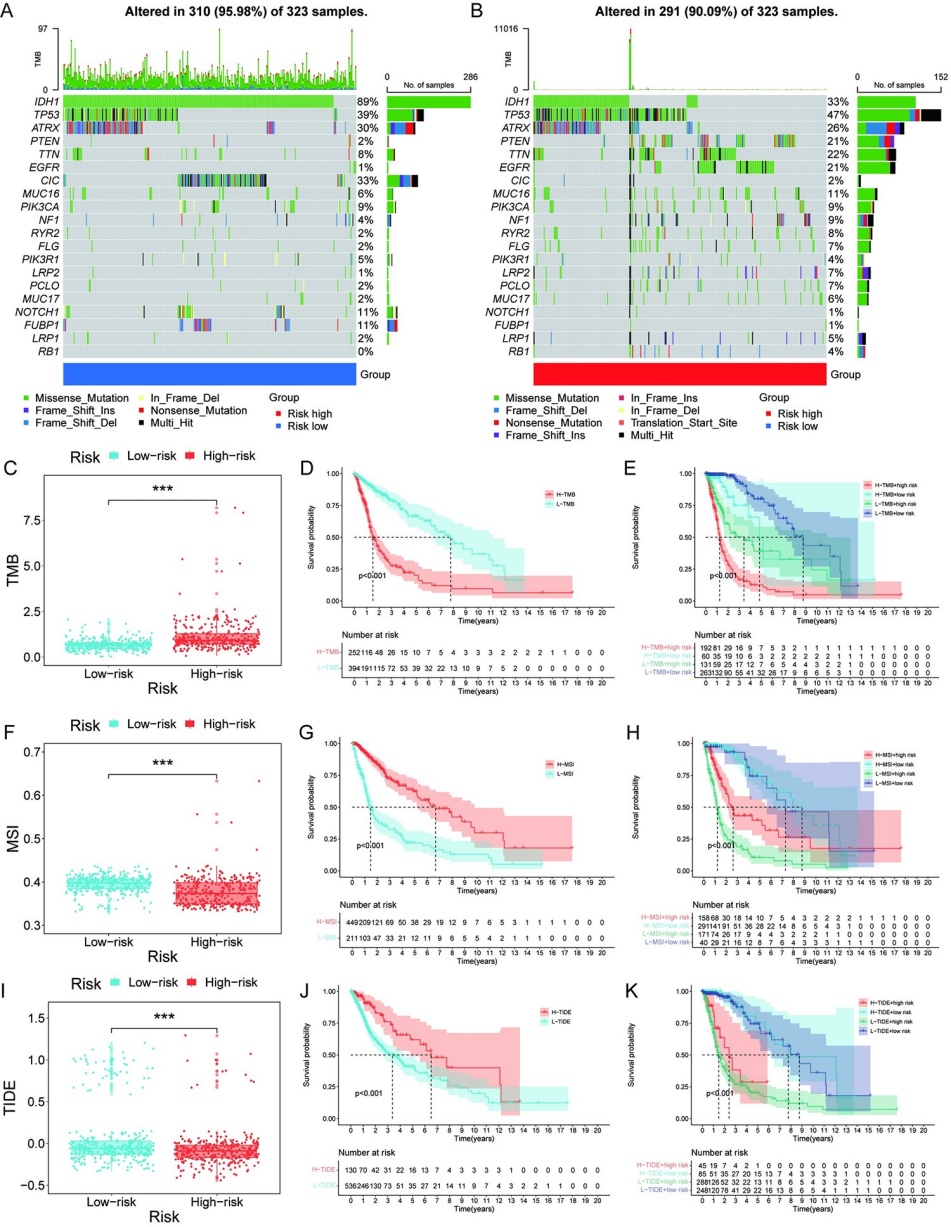
Mutational burden and microsatellite instability analysis of CDCAs in glioma. **(A-B)** Waterfall plots of tumor mutational load analysis for CDCA low risk and high-risk groups. **(C)** Box plot of tumor mutation load in the low and high-risk groups of CDCA. **(D)** Survival curve shows that glioma patients with high tumor mutation load have a lower probability of survival. **(E)** Survival curves showed that patients with high tumor mutation burden in the high-risk group had the worst probability of survival, while patients with ground tumor mutation burden in the low-risk group had the highest probability of survival. **(F)** Microsatellite instability analysis showed that glioma patients in the high-risk group had more stable microsatellites, predicting poor immunotherapy efficacy in the high-risk group. **(G)** Survival curve demonstrated that glioma patients with high levels of MSI had a higher probability of survival. **(H)** Patients with glioma at high risk accompanied by low levels of MSI had the lowest probability of survival compared to the other groups. **(I)** TIDE score analysis showed a high value of immunotherapy for glioma patients in the high-risk group. **(J)** Patients with lower TIDE scores for glioma had a lower probability of survival. **(K)** Patients at high risk with low TIDE scores had the lowest probability of survival. Significance markers: ***, p<0.001.

By contrast, patients in the low-risk cohort had higher frequencies of IDH1, CIC, NOTCH1, and FUBP1 mutations **(Fig 5A)**. Patients in the high-risk cohort had higher TP53, PTEN, TTN and EGFR mutations frequencies **(Fig 5B)**. Patients in the high-risk cohort revealed higher TMB levels **(Fig 5C)**. The results recommend that patients with high levels of TMB may have a more valuable immunotherapeutic potential. Further analysis suggested that glioma patients with higher levels of TMB had a poorer outcome **(Fig 5D)**. Patients with high-risk and high-TMB had the worst outcomes, and low-risk low-TMB patients had the best outcomes in whole cohort **(Fig 5E)**. Gliomas in the high-risk cohort were interrelated with lower MSI levels **(Fig 5F)**. Patients with low MSI had a worse outcome than those with high MSI **(Fig 5G)**. Whereas, patients with high-risk and low-MSI had the worst survival in the whole cohort **(Fig 5H)**. The results were suggestive that the high-risk cohort was interrelated with lower MSI levels and was interrelated with a lower probability of survival for glioma. Low levels of MSI may also be connected to poor prognosis after glioma surgery. Gliomas in the high-risk cohort were interrelated with lower scoring of TIDE **(Fig 5I)**. Compared with high TIDE, the prognosis of patients with low TIDE is worse **(Fig 5J)**. Patients with high risk and low TIDE score have the poorest outcomes **(Fig 5K)**. The low TIDE scores in the high-risk cohort also suggest a higher benefit of immunosuppressive blockade therapy in glioma patients.

### Glioma clusters based on CDCAs had distinct functions and pathway activities

**Fig 6:**
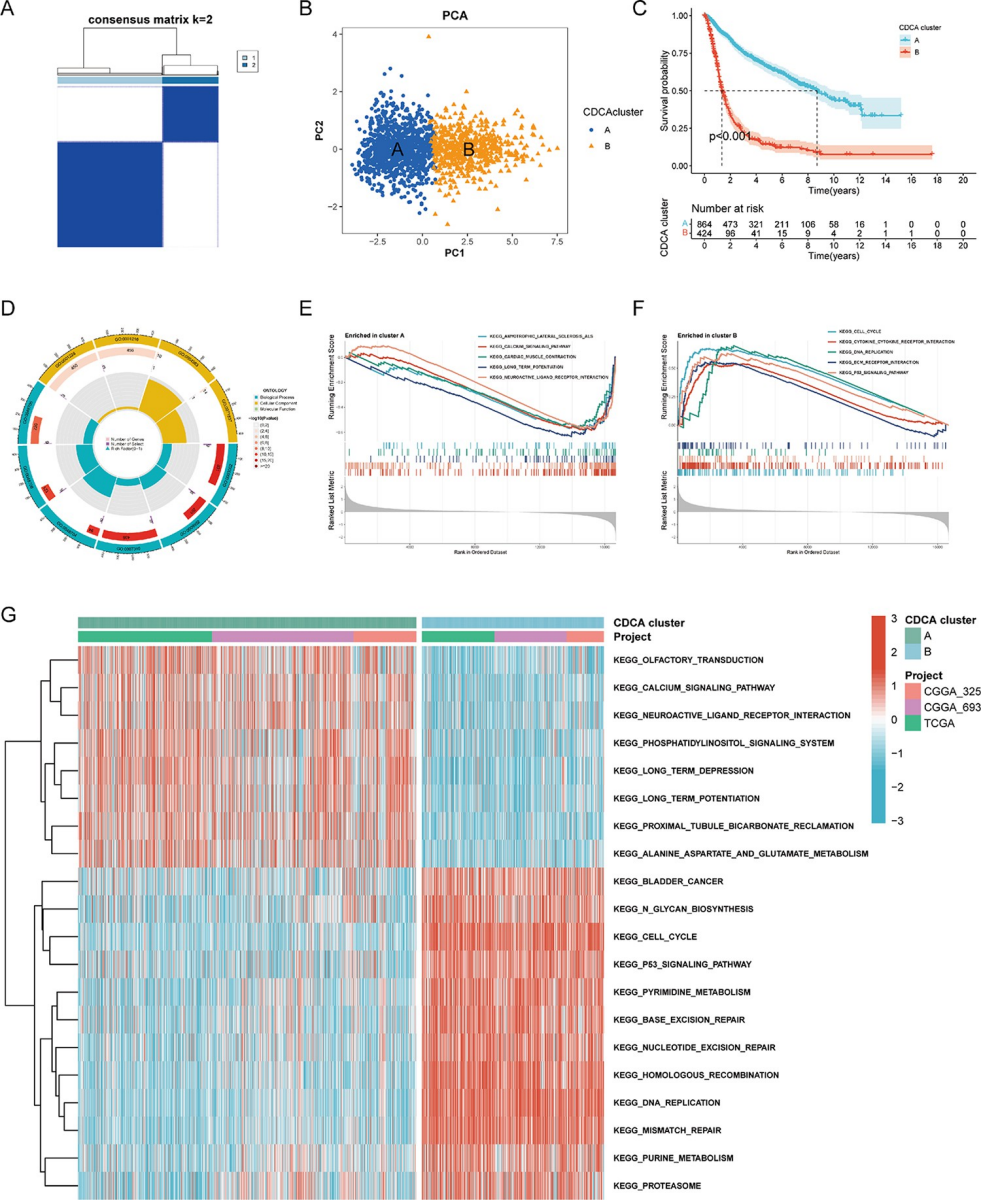
Glioma clusters based on CDCAs had distinct functions and pathway activities. **(A)** Consensus matrix heatmap showing glioma samples classified into two related clusters. **(B)** Plot of principal components analysis. Blue dots represent cluster A, yellow triangles represent cluster B. **(C)** Kaplan-Meier curves of two clusters. Red represents cluster B and blue represents cluster A. **(D)** Circle plot of GO enrichment analysis. The color of the outermost circle and innermost sector represents the type of ontology, blue represents biological processes (BP), yellow represents cellular components (CC), and green represents molecular functions (MF). The ring color of the second outer circle represents the -log(pvalue) of the pathway, and the length represents the number of genes in the pathways. The height of the innermost sector represents the enrichment factor which is from 0 to 1. The purple ring in the second inner circle represents the number of genes selected by the enrichment pathways. **(E)** GSEA analysis of two clusters that normalized enrichment score (NES) are negative. **(F)** GSEA analysis of two clusters that NES are positive. **(G)** KEGG pathway heatmap of GSVA analysis. Red represents high expression of pathway gene sets in glioma samples, and blue represents low expression.

According to the optimal result of the consensus clustering algorithm, we classified the merged gliomas samples of TCGA and 2 CGGA sets into 2 clusters of CDCAs, cluster A and B **(Fig 6A)**. The CDCAs clustering had favorable discrimination ability for sample distribution **(Fig 6B)**. We estimated the expression of CDCAs in the two clusters, and cluster B had higher CDCAs expression than cluster A. Subsequently, we actualized a survival analysis on the CDCAs clusters. Clusters B patients had worse OS (log rank p < 0.001) **(Fig 6C)**. GO analysis revealed that the differentially expressed genes between the clusters were mainly involved BP in pattern specification process and embryonic skeletal system morphogenesis, and involved MF in DNA-binding transcription activator activity and RNA polymerase II-specific **(Fig 6D)**. GSEA analysis presented that pathways active in cluster A were neuroactive ligand receptor interaction and calcium signaling pathway **(Fig 6E)**. The pathways active in cluster B were ECM receptor interaction and cytokine receptor interaction **(Fig 6F)**. GSVA analysis presented that the pathways activated in cluster A samples included alanine aspartate and glutamate metabolism and long-term depression, the pathways activated in cluster B samples included cell cycle and base excision repair **(Fig 6G)**.

### Cluster-based analysis reveals that glioma samples in cluster B have a higher proportion of immune cells and are interrelated with modulation of the immune profile

**Fig 7:**
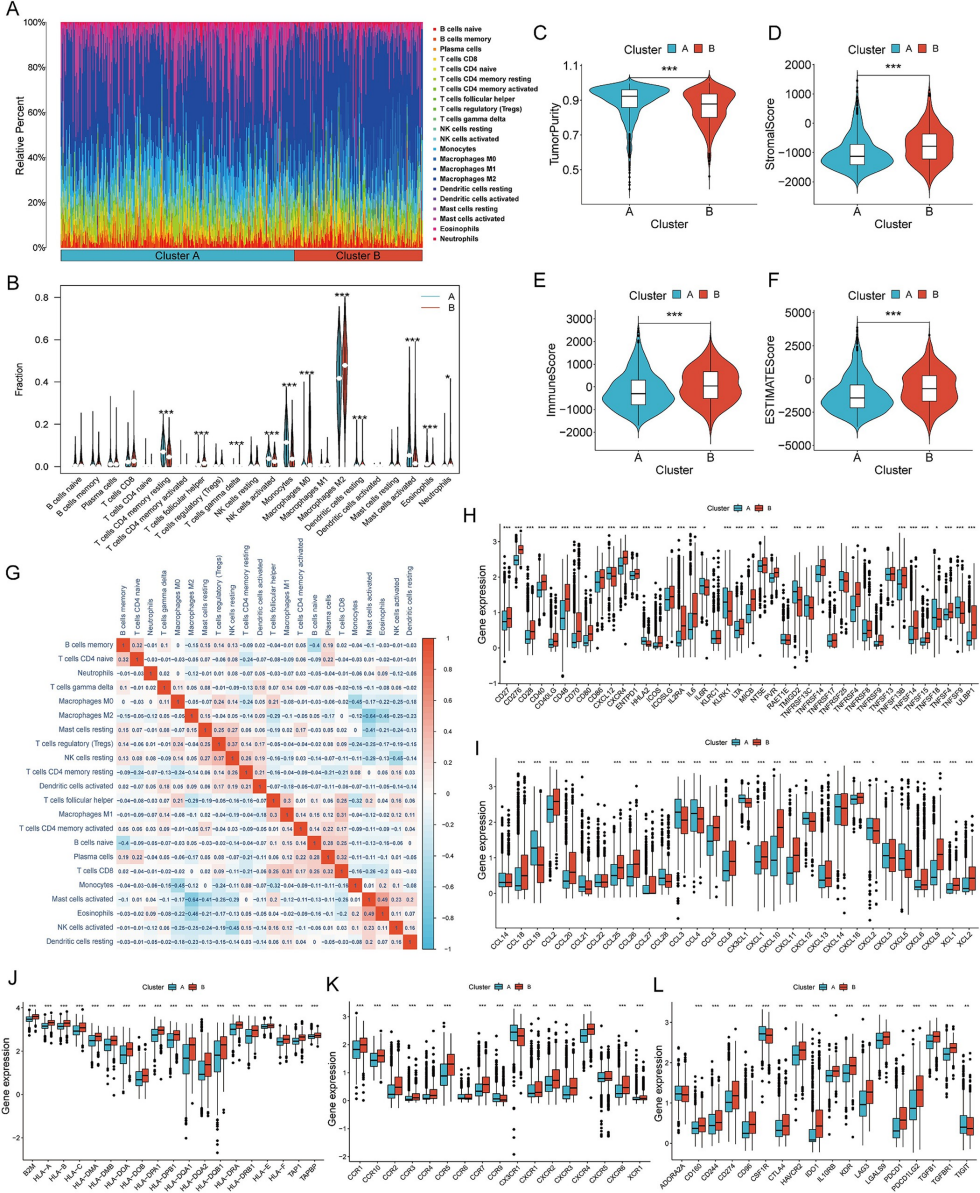
Glioma samples of cluster B had a higher proportion of immune cells and immune escape ability. **(A)** Barplot of 22 immune cells proportion in two clusters. Each different color represents a type of immune cell. **(B)** Violin plot of the difference in immune cell proportions between the two clusters. Red represents cluster B, and blue represents cluster A. **(C-F)** Box plots of immune landscape analysis based on ESTIMATE algorithm, including tumor purity **(C)**, stromal score **(D)**, immune score **(E)**, ESTIMATE score **(F)**. **(G)** Immune cell correlation heatmap based on CIBERSORT algorithm. Red represents positive correlation, and blue represents negative correlation. **(H-L)** Differential analysis between two clusters with respect to immunostimulatory **(H)**, chemokines **(I)** MHC molecules **(J)**, chemokine receptors **(K)**, immune checkpoints **(L)**.

Analysis of the proportion of immune cells showed that the macrophages M2 accounted for the highest proportion in all glioma samples **(Fig 7A)**. Analysis of differences in immune cell proportions revealed that NK cells activated, monocytes, mast cells activated, eosinophils had higher proportion in cluster A, and macrophages M2 and M0 had higher proportion in cluster B **(Fig 7B)**. Immune landscape analysis showed that cluster B had higher stromal score **(Fig 7C)**, immune score **(Fig 7D)**, and ESTIMATE score **(Fig 7E)**, but lower tumor purity **(Fig 7F)**. Immune cell correlation analysis presented a significant negative correlation between mast cells and macrophage M2, and a positive correlation between mast cells and eosinophils **(Fig 7G)**. We also measured the immune genes level with different functions **(Fig 7H–7L)**. Except for HHLA2, KLRC1, TMIGD2, and TNFSF9, most of immune-stimulator were upregulated in cluster B **(Fig 7H)**. Except for CCL3, CCL4, CCL19, and CXCL5, most of chemokine were higher expressed in cluster B **(Fig 7I)**. All of MHC molecules were highly expressed in cluster B **(Fig 7J)**. Except for CX3CR1, most of the chemokine receptor were upregulated in cluster B **(Fig 7K)**. Except for CSF1R, most of immune checkpoint were upregulated in cluster B **(Fig 7L)**. The study demonstrated that cluster B was not only associated with a high percentage of macrophage M2, but also with high antigen-presenting ability, chemokine regulation, and immune escape of tumor cells.

### Non-coding RNA-mediated regulation of CDCAs

**Fig 8:**
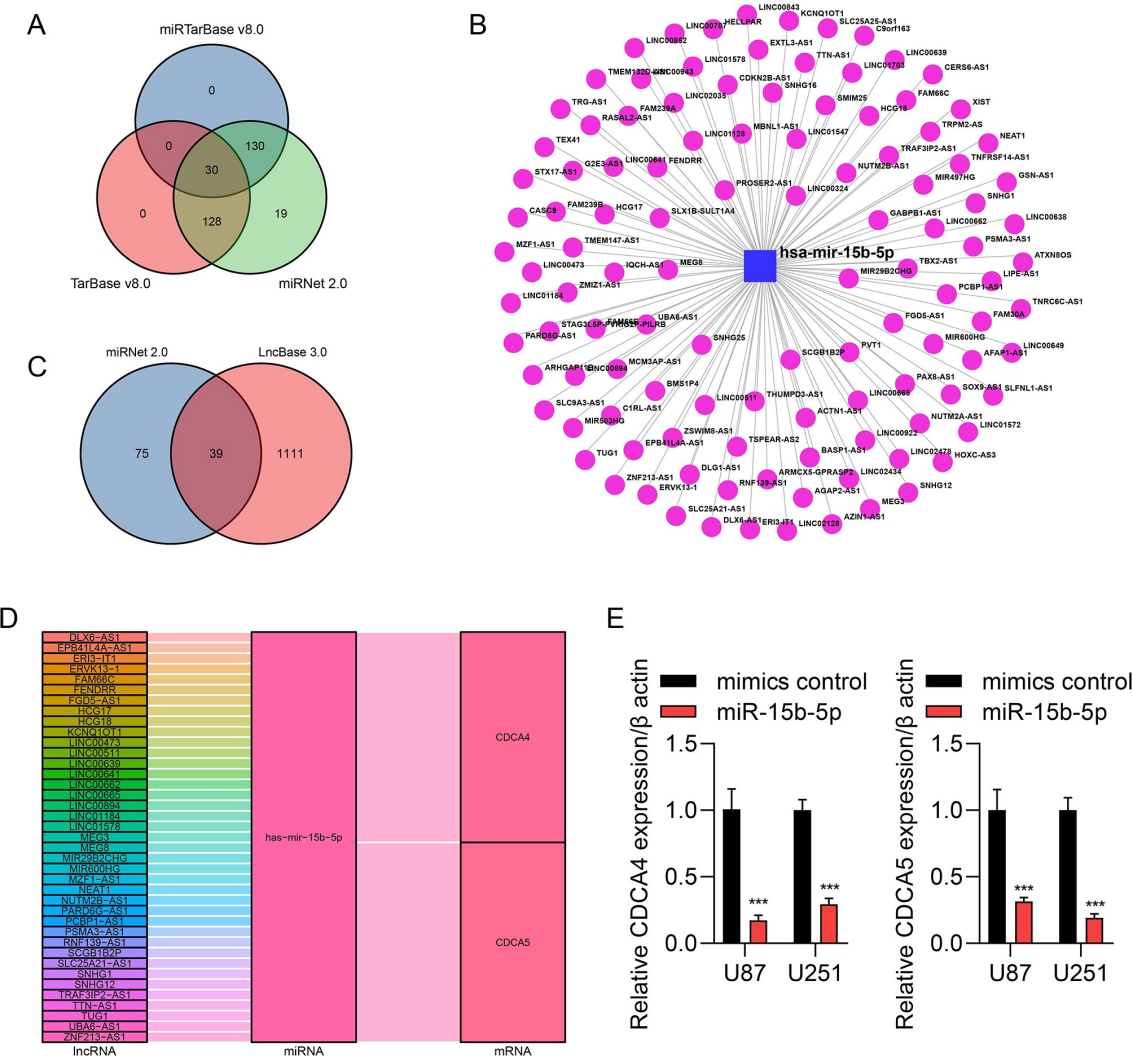
Screening for non-coding RNAs regulating CDCAs. **(A)** Venn plot showing miRNAs common to the miRTarbase, Tarbase, and miRNet databases. **(B)** Venn plot showing lncRNAs common to miRNet and LncBase databases. **(C)** lncRNAs related to hsa-mir-15b-5p (miRNet database). **(D)** Sankey plot showing that non-coding genes regulate CDCA4 and CDCA5. **(E)** qRT-PCR analysis of CDCA4 and CDCA5 in U87 and U251 cells transfected with mimics NC, or mimics miR-15b-5p. The expression of CDCA4 and CDCA5 was adjusted to the expression of β-actin. ***p < 0.001.

We used miRTarBase v8.0 **(S1A Fig)** and TarBase v8.0 **(S1B Fig)** in the NetworkAnalyst database, and miRNet 2.0 database **(S1C Fig)** to search for miRNAs related to CDACs and found that there were 30 miRNAs common to the three databases **(Fig 8A)**. MiRNet 2.0 database and LncBase 3.0 were further used to find upstream lncRNAs that could regulate miRNAs **(Fig 8C)**. Hsa-mir-26b-5p was identified from intersecting miRNAs using a Venn diagram **(Fig 8B)**. The miRNA-seq data of glioma from the TCGA database were combined with clinical data to find the miRNAs most associated with clinical characteristics. We found that hsa-mir-15b-5p was interrelated with WHO grade **(S2A Fig)**, age **(S2B Fig)**, IDH status **(S2C Fig)**, and 1p/19q codeletion **(S2D Fig)** in patients with glioma. Our analysis resulted in 39 lncRNAs related to hsa-mir-15b-5p, and a Sankey plot was constructed to show the corresponding competing endogenous RNA network **(Fig 8D)**. Moreover, the qRT-PCR assay indicated that miR-15b-5p significantly inhibited the expression of CDCA4 and CDCA5 in both cell types **(Fig 8E).**

### Drug sensitivity analysis

**Fig 9:**
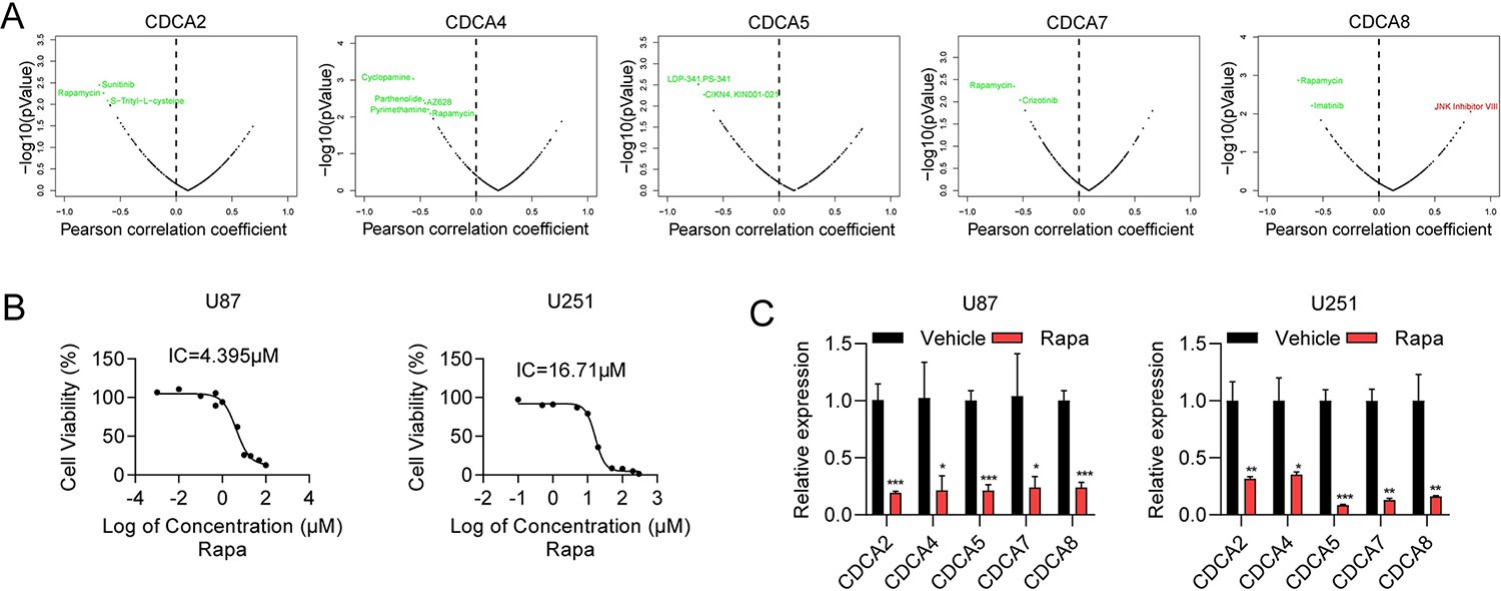
Drug sensitivity analysis of CDCAs. **(A)** Analysis of potential clinical therapeutic agents targeting for CDCA2, CDCA4, CDCA5, CDCA7, and CDCA8 (GDSC database). **(B)** The IC50 of Rapamycin in U87 and U251 cells detected by CCK8 assay. **(C)** qRT-PCR analysis of CDCA2, CDCA4, CDCA5 CDCA7 and CDCA8 in U87 and U251 cells treated with vehicle or rapamycin. The expression of indicated genes was adjusted to the expression of β-actin. Significance thresholds: ns, p ≥ 0.05; *p < 0.05; **p < 0.01; ***p < 0.001.

Combined with the GDSC drug database, CDCA2, CDCA4, CDCA5, CDCA7, and CDCA8 have potential as clinical drug treatments for glioma **(Fig 9A)**. Drug sensitivity analysis of CDCA3 was not queried in the GDSC drug database. Rapamycin was a potential therapeutic agent common to the CDCA family. To validate the impact of rapamycin on CDCA expression, we detected CDCA expression after rapamycin treatment. The IC50 of rapamycin was detected by CCK8 assay **(Fig 9B)**. We also found that rapamycin treatment significantly inhibited the expression of CDCA2, CDCA4, CDCA5, CDCA7, and CDCA8 in U87 and U251 cells **(Fig 9C)**. The CDCA family can be used as a drug sensitivity marker for rapamycin in the treatment of glioma.

## Discussion

The expression of CDCA mRNA was different in many cancer types from normal tissues. One of the characteristics of cancer development is proliferation. CDCA family genes play an important part in cell proliferation and have been deemed to be interrelated with recrudesce and worse prognosis in a variety of cancers [63]. In our analysis, CDCA 2, 3, 4, 5, 7, and 8 were significantly upregulated in glioma samples compared with normal samples, suggesting the potential effects.

CDCAs were also significantly interrelated with clinical characteristics of patients with glioma. All CDCAs showed good clinical correlation with WHO grade and histological type. As the malignant degree of glioma increases, the expression of CDCA family is up-regulated. CDCA2, 4, 8 also had good clinical correlations with IDH status. Moreover, we found that CDCA8 not only had significant clinical correlations with important clinical features of glioma patients, including WHO grade, IDH status, and 1p/19q codeletion, but also had good diagnostic efficacy. To further refine the expression pattern of CDCA family in glioma, we actualized single-cell analysis. The CDCA family was specifically expressed in malignant glioma cells, while CDCA7 is specifically expressed in NPC-like malignant cells. Tirosh et al. identified a subgroup of undifferentiated cells in oligodendroglioma with a neural progenitor/stem cell expression program [64]. This NPC-like cell compartment is enriched in cycling cells and is the primary source of proliferating cells in oligodendrogliomas. This expounded a potential mechanism of CDCA7 in regulating the proliferative ability of glioma.

CDCAs have extremely important prognostic value in patients with glioma. We performed survival analysis and found that CDCA 2, 3, 4, 5, 7, and 8 were related to worse prognoses in glioma patients. We validated this result with multiple databases. However, CDCA5 did not present any prognostic effect in the GEO dataset. Some members of the CDCA family may be the prognostic factors for glioma patients. Cox regression analysis indicated that CDCA 2, 5, and 8 were independent prognostic factors for glioma. We therefore believe that CDCAs have high prognostic value in glioma. To further analyze this prognostic value, we used LASSO regression, a machine learning algorithm, to fit a gene family risk model. The model constructed based on CDCA 2, 3, 4, 5, 7, and 8 showed high diagnostic ability. Moreover, compared with the individual CDCA gene, the model showed better predictive ability, which has potential clinical application value. The prognostic ability of the model was verified in multiple external validation datasets.

To explore the source of the discrepancy in outcomes between two CDCA cohorts, we analyzed tumor immune escape and mutations. The results showed that high-risk cohorts had the characteristics of high TMB, low MSI, and low TIDE, and these characteristics predicted a poorer outcome for glioma patients. A large amount of research evidence has shown that tumor cel ls with high TMB have a higher antigen load, which leads to the body’s anti-tumor immune response [65, 66]. And this provides avenues for glioma immunotherapy [67].High TMB has become an effective marker of immune checkpoint blockade therapy [68]. As a marker for evaluating tumor immune escape, TIDE score is considered to be a more accurate signature for evaluating tumor immunotherapy than TMB [49]. TIDE score and TMB both reveal higher immunotherapy potential in high-risk cohorts. Numerous previous studies demonstrate better effect of anti-PD-1 immunotherapy for colorectal and prostate cancer patients with microsatellite instability [69, 70]. we demonstrated that high-risk cohorts had lower MSI scores. MSI was first used as a prognostic indicator for colorectal cancer, and MSI-H tumors are considered to have a better prognosis [71]. Later, MSI-stratified analyzes at the pan-cancer level indicated that gliomas were MSI-positive tumors and that low MSI scores predicted poorer prognosis in patients with low-grade gliomas [72]. The complex link between glioma immunotherapy and MSI still needs further exploration. However, there is currently no literature reporting that the CDCA family affects glioma TMB, MSI or immune escape at in vitro and in vivo levels. The underlying mechanism still needs further study.

To further search the mechanism of action of CDCA family in glioma, we separated glioma patients into two clusters. There was survival discrepancy between different CDCA clusters. GO analysis revealed that CDCA is mainly concentrated in DNA-binding transcription, and neuropeptide Y receptor activity. Further GSEA analysis presented that activated pathways in cluster B primarily include cell cycle, DNA replication and chemokine receptors. GSVA analysis also yielded similar results. Infinite proliferation is a characteristic of tumor cells. We therefore suggest that CDCA are engaged in the proliferation of tumor cells in glioma, and the regulatory effect is interrelated with the degree of malignancy. The cell cycle plays a crucial role in glioma progression, yet understanding is in its infancy [73]. Multiple glioma cell cycle regulators identified as potential therapeutic targets [74–76]. During the development of glioma stem cells, the CDCA family may be an important part of their proliferation regulation. In addition, CDCAs-focused chemokine receptors contribute to immune escape during glioma development [77]. This suggests that our CDCA family may be associated with the immune regulation of glioma.

Immunotherapy has gradually become a new treatment to eradicate cancer [78, 79]. CDCAs were deemed to be interrelated with most of immune cell types in our analysis, suggesting that CDCAs are interrelated with the glioma immune profile. The samples from Cluster B had lower tumor purity, suggesting that more non-tumor components were involved in the progression of gliomas in the CDCA high-expression cluster. Samples in cluster B had higher immune and stromal scores, as well as higher proportions of macrophages. This explains in part why the cluster B had poor prognosis. Microglia and macrophages are recruited by glioma cells and contribute to glioma growth and invasion [80]. The majority of the checkpoint, MHC molecule, chemokine, and chemokine receptor genes were expressed more strongly in the cluster B. This indicates that the high-CDCA cluster had greater antigen-presenting, directional chemotactic immune cell ability. According to the results of enrichment analysis, CDCA is more likely to act on the chemokine cascade to affect glioma progression. Most of the chemokines that have been shown to regulate glioma angiogenesis, such as CXCL9 and CXCL10, are highly expressed in cluster B [81]. While CX3CL1 and its receptor CX3CR1 are low expressed in cluster B, which have been proven to negatively regulate glioma invasiveness [82]. These results suggest that CDCA affects the immune microenvironment to promote the underlying mechanism of glioma progression.

We further analyzed upstream regulators of CDCAs. Analysis using multiple databases confirmed a total of 30 miRNAs associated with CDCAs. Combining these results with further clinical analysis, we found that hsa-mir-15b-5p was the most relevant miRNAs. Overexpression of hsa-mir-15b-5p was also confirmed via qRT-PCR analysis to suppress the expression of CDCA3 and CDCA4.

The IC50 values of rapamycin were found to show a negative correlation with CDCA expression. We suggested that rapamycin has the potential to regulate the expression of CDCAs, and further experiments verified that rapamycin treatment reduced the expression of CDCA 2, 4, 5, 7, and 8 in U87 and U251 cells. Rapamycin has high clinical application value.

To sum up, we created a prognostic model for glioma based on CDCAs. And further clustering to survey the mechanism of CDCA in glioma. High-risk cohorts have characteristics of high TMB, low MSI, and low TIDE. Glioma subtypes with high expression of CDCAs have stronger immune escape potential. We also found that CDCAs could be regulated by miRNA-15b-5p and that the expression of CDCAs may be correlated with rapamycin response. Thus, CDCAs have potential applications in clinical diagnosis and as drug sensitivity markers in gliomas. Our study provides new perspective for gene regulation and drug sensitivity in glioma, highlighting the potential clinical innovative applications of the CDCA family. Future research should focus on the specific pathways of CDCAs in glioma and the interaction between rapamycin and CDCAs.

## Supporting information

S1 Fig miRNAs related to CDCAs. **(A)** miRTarBase v8.0. **(B)** TarBase v8.0. **(C)** miRNet 2.0 database. Red, genes; blue, miRNAs.
(TIF)

S2 Fig Relationships between selected miRNAs and clinical characteristics of LGG patients. **(A)** WHO grade in patients with LGG. **(B)** IDH status in LGG patients. **(C)** Age in patients with LGG. **(D)** 1p/19q codeletion in LGG patients. Significance markers: ns, p≥0.05; *, p< 0.05; **, p<0.01; ***, p<0.001.
(TIF)
