# Supplementary figures and images for: Integrated bioinformatics analysis and experimental validation identified CDCA families as prognostic biomarkers and sensitive indicators for rapamycin treatment of glioma
Source: PLoS One. 2024 Jan 5;19(1):e0295346. doi: 10.1371/journal.pone.0295346 (PMC10769025; doi:10.1371/journal.pone.0295346)

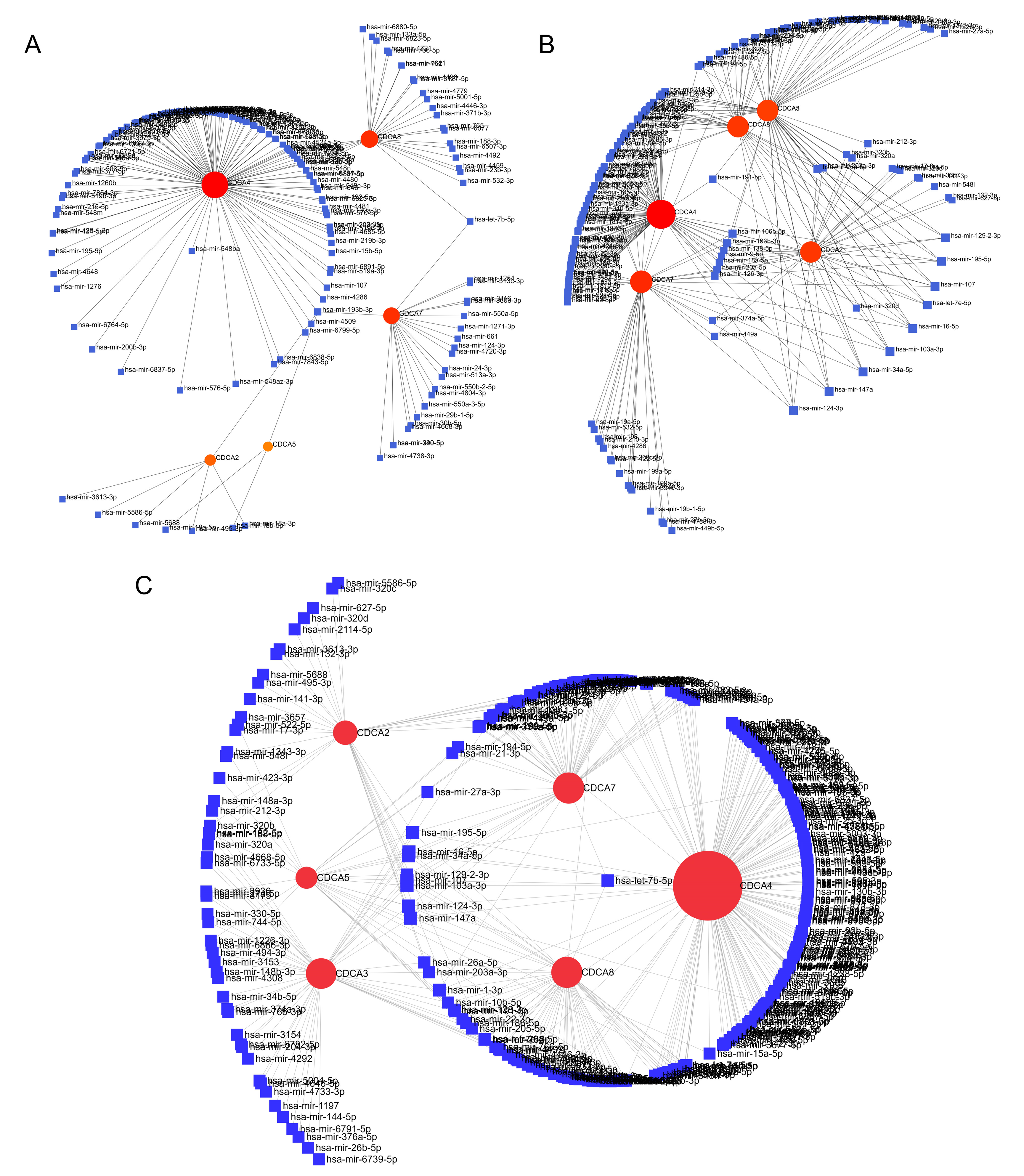

Supplement: S1 Fig — (A) miRTarBase v8.0. (B) TarBase v8.0. (C) miRNet 2.0 database. Red, genes; blue, miRNAs. (TIF) [file pone.0295346.s001.tif]

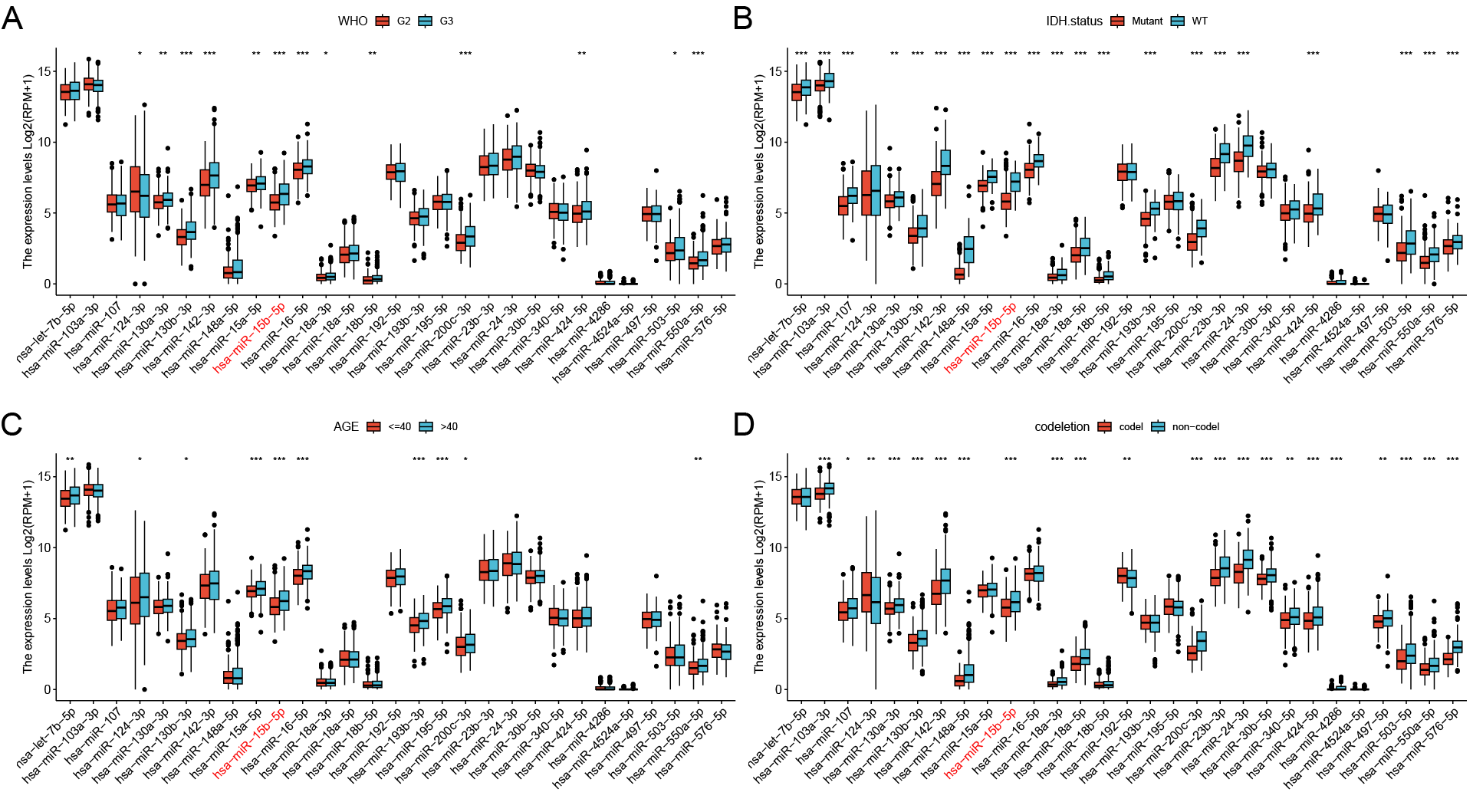

Supplement: S2 Fig — (A) WHO grade in patients with LGG. (B) IDH status in LGG patients. (C) Age in patients with LGG. (D) 1p/19q codeletion in LGG patients. Significance markers: ns, p≥0.05; *, p< 0.05; **, p<0.01; ***, p<0.001. (TIF) [file pone.0295346.s002.tif]
